# Genetic Heterogeneity among Chicken Infectious Anemia Viruses Detected in Italian Fowl

**DOI:** 10.3390/ani11040944

**Published:** 2021-03-27

**Authors:** Giulia Quaglia, Giulia Mescolini, Elena Catelli, Giacomo Berto, Filippo Muccioli, Caterina Lupini

**Affiliations:** 1 Department of Veterinary Medical Sciences, University of Bologna, 40064 Ozzano dell’Emilia, BO, Italy; giulia.quaglia2@unibo.it (G.Q.); elena.catelli@unibo.it (E.C.); caterina.lupini@unibo.it (C.L.); 2 CEVA Salute Animale, Via Bartolomeo Colleoni, 15, 20864 Agrate Brianza, MB, Italy; giacomo.berto.1@gmail.com; 3 Martini S.p.A., Via Emilia, 2614, 47020 Budrio di Longiano, FC, Italy; f.muccioli@martinigruppo.com

**Keywords:** chicken infectious anemia, Gyrovirus, immunosuppressive disease

## Abstract

**Simple Summary:**

Chicken infectious anemia virus (CIAV) is an immunosuppressive pathogen of chickens. In the present study field, vaccine-derived CIAV strains were reported to circulate in different types of chicken flocks. Viruses were successfully obtained from non-invasive samples such as feathers and environmental dust. Genome analysis showed that strains had heterogeneous sequences clustered into different genogroups that possessed genetic markers reported to be correlated with CIAV virulence. This survey contributes to the knowledge of field CIAV distribution maps and increases the existing information available on native isolates around the world.

**Abstract:**

Chicken infectious anemia virus (CIAV) is a pathogen of chickens associated with immunosuppression and with a disease named chicken infectious anemia. The present survey reports an epidemiological study on CIAV distribution in Italian broiler, broiler breeder and backyard chicken flocks. Twenty-five strains were detected by a specifically developed nested PCR protocol, and molecularly characterized by partial VP1 gene or complete genome sequencing. Viral DNA amplification was successfully obtained from non-invasive samples such as feathers and environmental dust. Sequence and phylogenetic analysis showed the circulation of field or potentially vaccine-derived strains with heterogeneous sequences clustered into genogroups II, IIIa, and IIIb. Marker genome positions, reported to be correlated with CIAV virulence, were evaluated in field strains. In conclusion, this is the first survey focused on the molecular characteristics of Italian CIAVs, which have proved to be highly heterogeneous, implementing at the same time a distribution map of field viruses worldwide.

## 1. Introduction

Chicken infectious anemia virus (CIAV), the only member of the genus *Gyrovirus* in the family *Anelloviridae* [1], is the causative agent of chicken infectious anemia (CIA) [2]. The CIAV genome is a negative sense, single-stranded, closed-circular DNA of approximately 2300 nucleotides in length [3,4], possessing three overlapping open reading frames (ORFs) [3] that encode three viral proteins of different molecular weights: VP1 (51.6 KDa), VP2 (24 KDa) and VP3 (13.6 KDa) [5]. Among these viral proteins, VP1, the major capsid protein, induces neutralizing antibody production in the host [3,6]. The non-structural VP2 is a dual-specific protein phosphatase that acts as a scaffolding protein to assist the correct conformation of VP1 [7,8]. Another non-structural protein, VP3, also known as apoptin, induces apoptosis in chicken thymocytes and lymphoblastoid T cells [9,10]. The VP1 gene is involved in viral replication and pathogenicity, and its hypervariable sequence has been widely investigated [11,12,13,14,15]. In contrast, VP2 and VP3 genes are more conserved among isolates [16]. Thus, for the genetic characterization of CIAV strains, the VP1 gene is primarily used [17]. Phylogenetically, based on the nucleotide sequence of the VP1 gene, four distinct genogroups/genotypes (I, II, III and IV) have been identified [18,19] and reported worldwide. Moreover, in the VP1 protein, there are vaccine genetic markers that allow the differentiation of the same from field strains [20]. The CIAV genome has been reported to undergo recombination events, which may have caused the emergence of novel genotypes [21].

CIAV is believed to be widespread in chicken producing countries; the course of the infection can be either clinical or subclinical [17]. The virus targets hemocytoblasts in the bone marrow and T lymphocytes in the thymus resulting in aplastic anemia, thrombocytopenia, leucopenia and thymus depletion [22]. Birds aged less than 2–3 weeks generally show anemia, runting and stunting, and increased mortality associated with bone marrow and thymus atrophy and subcutaneous hemorrhages observed at post mortem examination [23]. The disease can show different degrees of severity, depending, among other factors, on the immune status of the birds; namely, blood levels of maternally derived antibodies. The infection of chickens older than two weeks of age usually runs a subclinical course. Regardless of the clinical outcome, CIA is always associated with immunosuppression, often followed in the field by secondary infections with other pathogens and impaired immune responses to vaccinations [17]. CIAV-infected birds are likely to develop subcutaneous gangrenous dermatitis and secondary viral or bacterial infections of the respiratory tract [24,25].

Although CIA vaccination of broiler breeders is widely applied, ensuring control of the clinical disease in the offspring, a survey performed on 46 Italian broiler flocks showed serological positivity in 83% of these flocks at the slaughterhouse [26], suggesting that the virus is circulating in spite of the vaccination. However, no information is reported on the genetic characteristics of the strains.

In order to fill this gap, an epidemiological survey on CIAV distribution in Italian broiler, broiler breeder and backyard chicken flocks has been performed in the present study. The detected CIAV strains have been molecularly characterized by the VP1 gene or by complete genome sequencing.

## 2. Materials and Methods

### 2.1. Samples

Between 2017 and 2019, a total of 44 samples were collected and tested by PCR for the presence of CIAV. Samples originated from different farms and flocks of pullet broiler breeders (*n* = 11), broilers (*n* = 25) or backyard chickens (*n* = 8), located in different Italian regions and belonging to different poultry companies. Samples consisted of feathers and spleens collected from deceased birds or environmental dust, collected from the fans at the end of the production cycle (before or after the cleaning). Broiler breeder flocks were occasionally sampled twice.

Only pullet broiler breeders were vaccinated with live-attenuated vaccines against CIA (strain 26P4). In general, no overt clinical signs referable to CIA were observed in the considered flocks; in one case (sample 1196/19-farm 11), gangrenous dermatitis was reported. Details of the analyzed samples are reported in Table 1.

### 2.2. Sample Processing and DNA Extraction

DNA was extracted from samples using the commercial NucleoSpin^®^ Tissue kit (MACHEREY-NAGEL GmbH & Co. KG, Düren, Germany) according to the manufacturer’s instructions. Environmental dust was pretreated by resuspension of one gram of the sample in 5 mL of sterile 1× phosphate buffered saline (PBS), double centrifugation at 2500× *g* for 15 min at 4 °C, and filtration of the supernatant with a 0.45 µm syringe filter.

### 2.3. Nested-PCR

Extracted DNAs were screened for CIAV using a nested PCR protocol developed to specifically amplify the partial VP1 gene. Primers were designed on the genome sequence of the reference strain “Del-Ros” (GenBank accession no. AF313470) (Table 2) and used in PCR1 and PCR2, generating a final product of 790 bp (from nucleotide 996 to nucleotide 1786).

For each PCR reaction the following reaction mixture was prepared: 0.125 µL of GoTaq G2 Flexi DNA Polymerase 5 U/µL (Promega, Madison, WI), 5 µL of 5× Green Go-Taq^®^ Flexi Reaction Buffer (Promega, Madison, WI, USA), 1.75 µL of 25 mM MgCl_2_ solution, 0.5 µL of dNTPs (0.2 mM), 11.625 µL of H2O for molecular biology, 0.5 µL of each primer (0.2 µM) and 5 µL of the extracted DNA or PCR 1 amplicon. Cycling conditions were as follows: 2 min of denaturation at 94 °C followed by 35 cycles, each consisting of denaturation at 94 °C for 1 min, annealing at 50 °C for 1 min, and extension at 72 °C for 1.5 min. A final elongation step at 72 °C for 5 min completed the reaction. The PCR2 product was separated in 1.5% agarose gel, stained with MIDORI^green^ Advance (NIPPON Genetics Europe, Düren, Germany), and visualized under ultraviolet light.

### 2.4. Whole Genome Amplification and Sequencing

In order to amplify the whole genome of the detected CIAV strains, DNAs were subjected to three overlapping PCRs following the protocol reported by Li et al. [27]. The obtained amplicons were purified using ExoSAP-IT™ Express PCR Product Cleanup (Thermo Fisher Scientific, Massachusetts, MA, USA) following the manufacturer’s instructions, and sequenced in both directions by Macrogen Europe (Amsterdam, The Netherlands). When full amplification of the CIAV genome was not successful, strains were sequenced in their partial VP1 gene previously amplified.

### 2.5. Sequence and Phylogenetic Analysis

The obtained nucleotide sequences were assembled and edited using the Bioedit Sequence Alignment Editor, Version 7.2.5.0 (Tom Hall, Ibis Therapeutics, Carlsbad, CA, USA), then aligned and compared using Clustal W software [28], with homologous sequences of CIAV reference strains retrieved from the GenBank database (Table 3).

Phylogenetic trees, based on partial or complete VP1 gene nucleotide sequences, were generated with the neighbor-joining method, using MEGA X [38]. The branch support was calculated by performing 1000 bootstrap replicates; only branches supported by bootstrap values equal or greater than 70 were considered reliable. Highest nucleotide similarity with publicly available nucleotide sequences was determined using the Basic Local Alignment Search Tool (BLAST) [39].

### 2.6. Recombination Analysis

A dataset containing the obtained sequences and 88 additional complete CIAV genomes retrieved from GenBank (Table S1) was analyzed to identify putative homologous recombination events by the Recombination Detection Program 4 (RDP v.4.97) software suite [40]. The following detection methods implemented in the program were used: RDP, Geneconv, Bootscan, Maxchi, Chimaera, Siscan and 3Seq; the *p*-Value was adjusted to 0.05. Only recombination events supported by no fewer than five independent detection methods were regarded as positive.

### 2.7. Accession Numbers

Sequences obtained in this study were submitted to the GenBank database and are available under the following accession numbers: MT813068–MT813092.

## 3. Results

**Figure 1:**
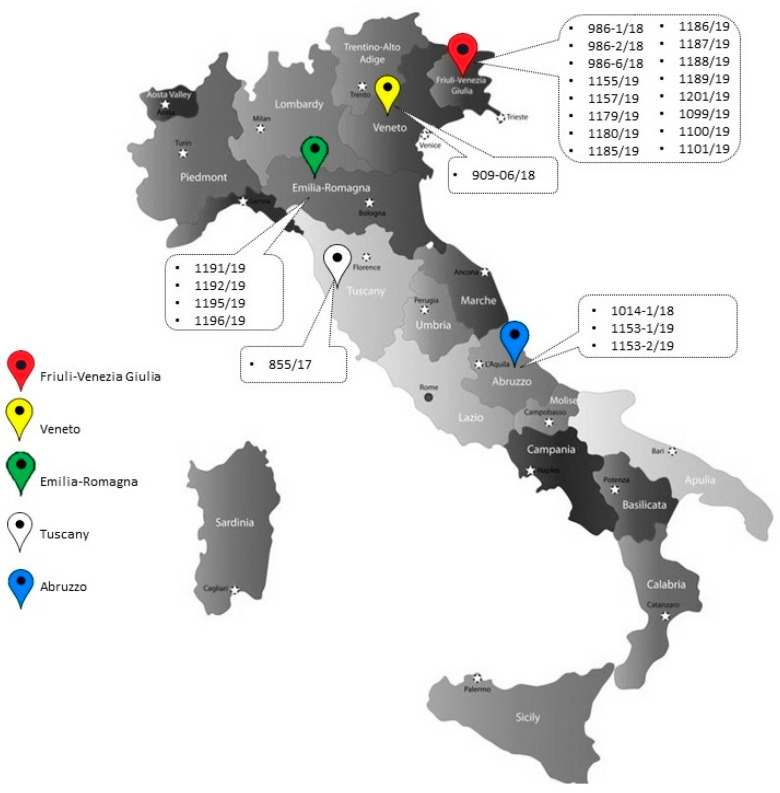
Distribution map of the CIAV strains detected in the study.

Twenty-five CIAV strains were detected by nested-PCR during the study and named using the following nomenclature: CIAV/country of origin (Italy = IT)/Host Species (Chicken = CK)/sample ID number/year (Table 1). A distribution map, showing the geographic distribution of the detected strains, is shown in Figure 1. Partial VP1 nucleotide sequences were obtained for all the detected strains; full genome sequencing was successful for 11 out of 25 strains. Results showed that 42.9% of breeder pullet, 84% of broiler and 12.5% of backyard chicken flocks tested positive for CIAV.

### 3.1. Phylogenetic Analysis

**Figure 2:**
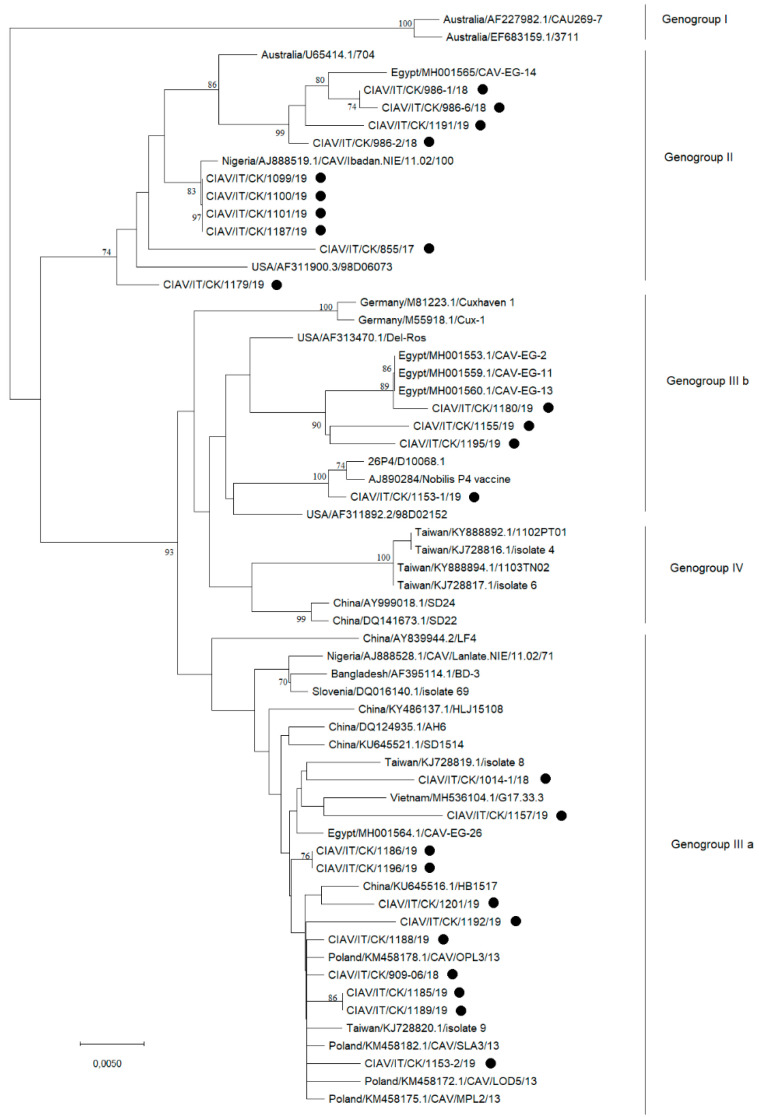
Phylogenetic tree based on the partial VP1 nucleotide sequence of twenty-five Italian CIAV strains (marked with a black circle) and reference CIAV strains retrieved from GenBank. Genogroup classification is also indicated on the right of the tree. Only bootstrap values ≥ 70 are reported. The tree is drawn to scale, with branch lengths in the same units as those of the evolutionary distances used to infer the phylogenetic tree.

The phylogenetic tree based on the partial VP1 nucleotide sequences of newly detected and reference CIAV strains is shown in Figure 2.

CIAV strains are classified into genogroups I, II, IIIa and IIIb [18] and IV [19], and the Italian strains analyzed in the present study belonged to genogroups II (n.10 strains), IIIa (n.11 strains) or IIIb (n.4 strains). Noticeably, the CIAV/IT/CK/1153-1/19 strain clustered with the vaccine strain 26P4, and strains CIAV/IT/CK/1155/19, CIAV/IT/CK/1180/19 and CIAV/IT/CK/1195/19 with the Del-Ros vaccine strain, both in genogroup IIIb.

**Figure 3:**
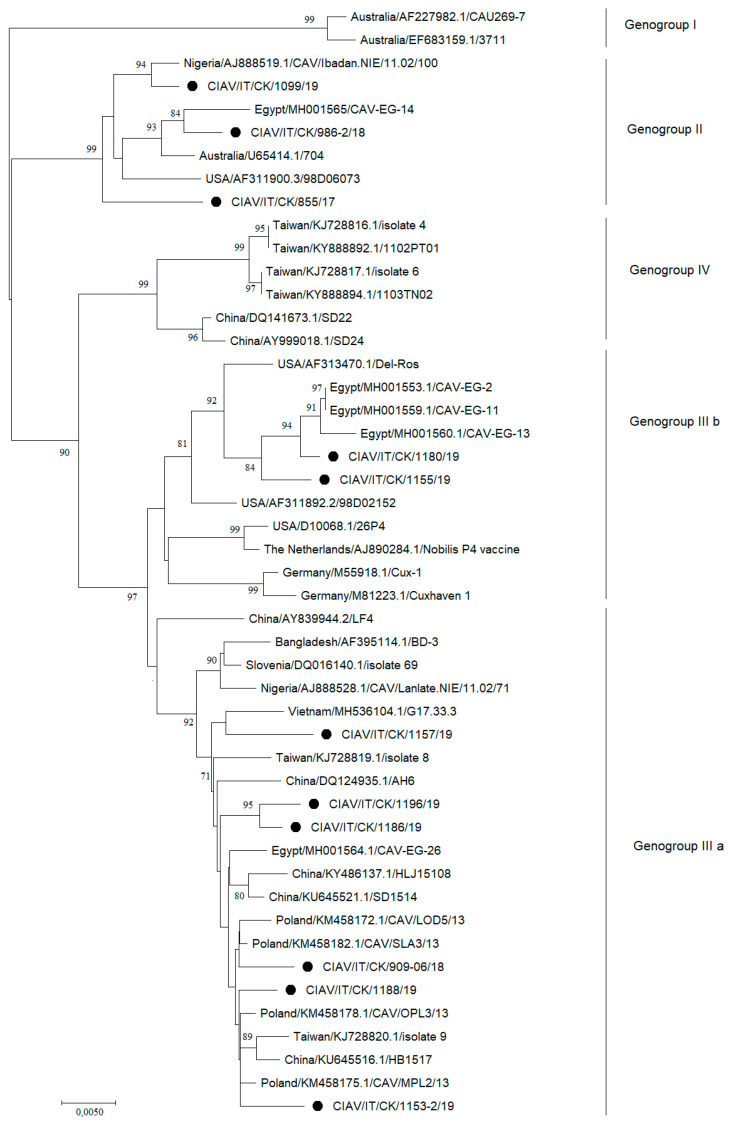
Phylogenetic tree based on complete VP1 nucleotide sequences of eleven Italian CIAV strains (marked with a black circle) and reference CIAV strains retrieved from GenBank. Genogroup classification is also indicated on the right of the tree. Only bootstrap values ≥ 70 are reported. The tree is drawn to scale, with branch lengths in the same units as those of the evolutionary distances used to infer the phylogenetic tree.

Bootstrap values indicated on the branches that define genogroups IIIb, IV, and IIIa were less than 70. In order to verify the division into these genogroups, a phylogenetic analysis including the 11 complete VP1 sequences obtained and the reference sequences available on GenBank was performed and confirmed the Italian strains classification with bootstrap values > 70 (Figure 3).

### 3.2. Sequence Analysis

The Italian CIAV strains showed between their VP1 gene sequences a percentage of identity ranging from 94% to 100% or from 96% to 100% at the nucleotide or amino acid level, respectively. Moreover, the majority of the strains (21 out of 25) had >99% nucleotide identity with field strains detected in other countries. In agreement with phylogenetic results, in the partial VP1 sequence, strain CIAV/IT/CK/1153-1/19 had 99.5% nucleotide sequence identity with the 26P4 vaccine strain, and strains CIAV/IT/CK/1155/19, CIAV/IT/CK/1180/19 and, CIAV/IT/CK/1195/19 had 97.8% mean nucleotide sequence identity with the Del-Ros vaccine strain.

The patterns of amino acid substitutions in specific amino acid sites, located at positions 22, 75, 97, 125, 139, 144, 157, 287, 290, 370, 394 and 413 of the VP1 protein, are reported in Table 4. These amino acids showed variability among CIAV isolates [19,21,35,41], and changes in some of them are associated with viral attenuation [11,12,13,42,43,44].

The full genome sequencing allowed us to analyze VP2 and VP3 genetic variability among the detected strains, and in comparison with other strain sequences retrieved from GenBank. The VP2 and VP3 nucleotide and amino acid sequences were highly conserved, both showing a mean percentage of identity between the analyzed strains of 99.7% and 100%, respectively. Moreover, full genome sequencing of the strains CIAV/IT/CK/1155/19 and CIAV/IT/CK/1180/19 showed 98.8% mean nucleotide sequence identity with the Del-Ros vaccine strain.

Recombination analyses showed that homologous recombination phenomena were not found in the obtained sequences.

## 4. Discussion

For the first time, in this study, the Italian epidemiology of CIAV in different poultry production types was investigated, and the detected strains were molecularly characterized. A total of twenty-five CIAV strains were detected by a newly developed nested-PCR protocol either from broiler, broiler breeder or backyard chickens. The method was effective in detecting the virus in both conventional or unusual matrices, such as feathers or environmental dust, known to harbor low viral titers [45]. Remarkably, these specimens are considered ethically acceptable, having been collected with non-invasive procedures, and can be used for the simultaneous detection of various avian infectious agents [46].

Based on sequence analysis, the detected strains were genetically characterized in marker positions reported to be correlated with CIAV biological features. Interestingly, 23 out of 25 Italian strains detected in this study showed in their VP1 proteins an identical amino acid motif composed of glutamines in positions 139 and 144. CIAV infectious clones, manipulated to introduce glutamine substitutions in these positions, have been reported to show a low in vitro pathogenic potential, documented by a decreased rate of replication and spread of the virus in cells than viruses with lysine and aspartic acid at these positions [11]. Chowdhury et al. [13] found that a change from glutamines to lysine and glutamic acid at these positions was associated with loss of virulence. Furthermore, experimental infection with a low-passage CIAV isolate with the glutamines pattern showed that it was pathogenic [47]. In order to definitively confirm the biological characteristics of the genetic profile found in our strains, additional in vivo pathogenicity studies should be performed in sensitive birds in secure isolation conditions.

Phylogenetic analyses based on VP1 genes showed that the detected strains are included in different genogroups, indicating molecular heterogeneity of the CIAV strains circulating in Italy. The majority of the Italian viruses belong to genogroups II and IIIa, along with field strains detected in different geographic areas of the world.

The strain CIAV/IT/CK/1153-1/19, detected from environmental dust collected from a farm housing 16-week-old CIAV vaccinated broiler breeders, clustered into genogroup IIIb together with the vaccine strain 26P4, suggesting the environmental persistence of the vaccine virus used in Italy. Furthermore, strains CIAV/IT/CK/1180/19, CIAV/IT/CK/1155/19 and CIAV/IT/CK/1195/19, clustering with the Del-Ros vaccine strain and having 98.8% mean nucleotide sequence identity in the VP1 gene with the Del-Ros vaccine strain, were interestingly detected in non-vaccinated broilers. Detection of strains with sequences close to the Del-Ros vaccine has been previously reported in Egyptian broilers [34], allowing us to suppose that a vaccine-derived strain or a field strain with a sequence close to this vaccine can circulate. Commercially available CIA vaccines are derived from field strains serially passaged in cells or chicken embryos for attenuation [48]. However, the level of attenuation does not usually prevent vertical or horizontal transmission of the vaccines to and between offspring [49]. This vaccine behavior could consequently be a hazard for young chicks, since it has been demonstrated that attenuated CIA strains have the potential to revert to virulent phenotypes after chicken-to-chicken transmission in the field [50,51]. Vaccine persistence, circulation and reversion to virulence have also been frequently reported for other empirically attenuated avian vaccines developed to control immunosuppressive or respiratory diseases [52,53,54]. Next-generation vaccines, with improved stability and safety, are currently under study and development for most avian pathogens and, in the future, they will likely replace traditional vaccines [55,56,57,58].

Looking at our results, it is furthermore possible to observe that different genogroups can circulate in the same broiler farm, possibly due to multiple breaches in the applied biosecurity measures. This hypothesis is supported by the circulation of strains belonging to the same genogroup II, both in commercial broiler flocks and in backyard chickens. The potential role of backyard chickens as a reservoir of avian pathogens for intensive breeding has been repeatedly emphasized, especially when they are located in densely poultry-populated areas [59,60,61,62]. In our study, CIAV field strains have also been detected in pullet broiler breeders, indicating that the circulation of this virus is also possible when vaccination and high biosecurity measures are in place.

Although no overt clinical signs have been observed in most of the investigated flocks, co-infection with Marek’s disease virus and infectious bursal disease virus has been reported in three of the flocks found positive for CIAV; multiple infection with immunosuppressive agents has been previously reported, and this scenario can further impair the immune response of the host [46,63]. An outbreak of gangrenous dermatitis correlated with the isolation of Clostridium spp. was reported in broiler chickens of farm 11, found infected with a CIAV strain genetically identical (100% nucleotide identity) to another strain detected in a healthy flock. The presence of secondary bacterial infections in CIAV immunosuppressed birds is in fact influenced by various factors such as the age at infection and the environmental virus load [48].

## 5. Conclusions

In conclusion, this survey, relying on VP1 and full genome sequencing, is the first to focus on the molecular characteristics of CIAV strains circulating in Italy. Our data contribute to the knowledge of field distribution maps of chicken anemia virus and increase the existing information available on native isolates around the world.

## Figures and Tables

**Table 1:** Details of the samples collected and results of chicken infectious anemia virus (CIAV) detection and genogrouping.

Production Type	Sample ID	Sample	Farm	Flock	Italian Region	Poultry Company	Age of Birds/Time of Sampling	CIAV PCR Results (Genogroup)
Broiler	909-06/18 **	Feathers *	1	A	Veneto	1	40 days	Positive (IIIa)
	986-1/18	Dust	2	A	Friuli-Venezia Giulia	2	End of production cycle (post-cleaning)	Positive (II)
	986-2/18 **			B				Positive (II)
	986-6/18			C				Positive (II)
	1018/18	Dust	3	A	Friuli-Venezia Giulia	2	End of production cycle (post-cleaning)	Negative
	1019/18			B				Negative
	1155/19 **	Dust	4	A	Friuli-Venezia Giulia	2	End of production cycle (before-cleaning)	Positive (IIIb)
	1157/19 **	Feathers		B				Positive (IIIa)
	1178/19	Dust	5	A	Friuli-Venezia Giulia	2	N.A.	Negative
	1179/19			B				Positive (II)
	1180/19 **			C				Positive (IIIb)
	1185/19	Dust	6	A	Friuli-Venezia Giulia	2	N.A.	Positive (IIIa)
	1186/19 **			B				Positive (IIIa)
	1187/19			C				Positive (II)
	1188/19 **	Dust	7	A	Friuli-Venezia Giulia	2	N.A.	Positive (IIIa)
	1189/19			B				Positive (IIIa)
	1191/19	Dust	8	3,4,5,6	Emilia-Romagna	2	End of production cycle (before-cleaning)	Positive (II)
	1192/19			7,8,9				Positive (IIIa)
	1194/19	Dust	9	/	Emilia-Romagna	2	35 days	Negative
	1195/19	Dust	10	/	Emilia-Romagna	2	47 days	Positive (IIIb)
	1196/19 **	Spleen *	11	/	Emilia-Romagna	2	N.A.	Positive (IIIa)
	1201/19	Dust	12	/	Friuli-Venezia Giulia	2	42 days	Positive (IIIa)
	1099/19 **	Dust	13	A	Friuli-Venezia Giulia	3	End of production cycle (post-cleaning)	Positive (II)
	1100/19			B			End of production cycle (post-cleaning)	Positive (II)
	1101/19			C			End of production cycle (before-cleaning)	Positive (II)
Pullet Broiler Breeders	989/18	Dust	14	A	Abruzzo	4	Day 0	Negative
	1014-1/18	Dust		B			12 weeks	Positive (IIIa)
	1152-1/19	Feathers *		C			16 weeks	Negative
	1153-1/19	Dust					16 weeks	Positive (IIIb)
	1218/19	Dust					End of production cycle (post-cleaning)	Negative
	1152-2/19	Feathers *		D			16 weeks	Negative
	1153-2/19 **	Dust					16 weeks	Positive (IIIa)
	1219/19	Dust					End of production cycle (post-cleaning)	Negative
	1220/19	Dust		E			End of production cycle (post-cleaning)	Negative
	1221/19	Dust		F			End of production cycle (post-cleaning)	Negative
	1015-2/18	Feathers *	15	/	Abruzzo	4	22 weeks	Negative
Backyard Chickens	801/17	Feathers *	/	G	Sicily	/	3.5 to 4 months	Negative
	810/17	Feathers *	/	H	Sicily	/	3 to 4.5 months	Negative
	847/17	Feathers *	/	I	Lombardy	/	12 months	Negative
	850/17	Feathers *	/	L	Tuscany	/	6 months	Negative
	852/17	Feathers *	/	M	Campania	/	6 to 9 months	Negative
	853/17	Feathers *	/	N	Lombardy	/	4 to 7 months	Negative
	854/17	Feathers *	/	O	Trentino-Alto Adige	/	9 to 24 months	Negative
	855/17 **	Feathers *	/	P	Tuscany	/	8 to 12 months	Positive (II)

/ not applicable, N.A. not available, * feathers and spleens were aseptically collected from 5 birds/flock and processed in pools, ** strain entirely sequenced.

**Table 2:** Oligonucleotide primers used for the nested-PCR.

PCR	PCR Primer Sequence 5′-3′	Position in the Genome	bp
**1**	5′-CAGGGTAAGCGAGCTAAAAG-3′	751–770	1528
	3′-GCTGCGTTTATTGAGTGC-5′	2262–2279	
**2**	5′-GGTACGTATAGTGTGAGGC-3′	996–1014	790
	3′-GCTGTGAGTGTTGCAAAGCT-5′	1767–1786	

**Table 3:** Reference strains retrieved from the GenBank database included in the analysis.

CIAV Strain	Country	GenBank Accession No.	Genogroup	Reference
Del-Ros	USA	AF313470	IIIb	N.A.
26P4	USA	D10068	IIIb	[29]
Nobilis P4 vaccine	The Netherlands	AJ890284	IIIb	[18]
Cux-1	Germany	M55918	IIIb	[3]
Cuxhaven 1	Germany	M81223	IIIb	[4]
CAU269-7	Australia	AF227982	I	[30]
3711	Australia	EF683159	I	N.A.
BD-3	Bangladesh	AF395114	IIIa	[31]
CAV/Ibadan.NIE/11.02/100	Nigeria	AJ888519	II	[18]
CAV/Lanlate.NIE/11.02/71	Nigeria	AJ888528	IIIa	[18]
69	Slovenia	DQ016140	IIIa	[32]
98D02152	USA	AF311892	IIIb	[33]
Isolate 4	Taiwan	KJ728816	IIIb	N.A.
Isolate 6	Taiwan	KJ728817	IIIb	N.A.
Isolate 8	Taiwan	KJ728819	IIIa	N.A.
Isolate 9	Taiwan	KJ728820	IIIa	N.A.
CAV-EG-2	Egypt	MH001553	IIIb	[34]
CAV-EG-11	Egypt	MH001559	IIIb	[34]
CAV-EG-13	Egypt	MH001560	IIIb	[34]
CAV-EG-14	Egypt	MH001565	II	[34]
CAV-EG-26	Egypt	MH001564	IIIa	[34]
CAV/LOD5/13	Poland	KM458172	IIIa	[35]
CAV/MPL2/13	Poland	KM458175	IIIa	[35]
CAV/OPL3/13	Poland	KM458178	IIIa	[35]
CAV/SLA3/13	Poland	KM458182	IIIa	[35]
G17.33.3	Vietnam	MH536104	IIIa	[36]
HB1517	China	KU645516	IIIa	N.A.
AH6	China	DQ124935	IIIa	N.A.
HLJ15108	China	KY486137	IIIa	[37]
LF4	China	AY839944	IIIb	N.A.
SD1514	China	KU645521	IIIa	N.A.
704	Australia	U65414	II	N.A.
98D06073	USA	AF311900	II	[33]
1102PT01	Taiwan	KY888892	IV	[19]
1103TN02	Taiwan	KY888894	IV	[19]
SD22	China	DQ141673	IV	N.A.
SD24	China	AY999018	IV	N.A.

**Table 4:** VP1 amino acid sequence comparison among the twenty-five Italian CIAV strains detected in the study and vaccine strains.

CIAV Strains	Amino Acid Position												
	22	75	97	125	139	144	157	287	290	370	394	413	447
Del-Ros	H	V	M	I	K	E	V	S	A	G	Q	S	G
26P4	.	.	.	.	.	.	M	T	.	S	.	A	T
CIAV/IT/CK/855/17	.	I	L	.	Q	Q	.	T	.	S	.	A	S
CIAV/IT/CK/909-06/18	.	I	L	.	Q	Q	.	A	.	T	.	A	S
CIAV/IT/CK/986-1/18	-	I	L	L	Q	Q	.	T	P	-	-	-	-
CIAV/IT/CK/986-2/18	.	I	L	.	Q	Q	.	T	P	T	.	A	S
CIAV/IT/CK/986-6/18	-	I	L	L	Q	Q	.	T	P	-	-	-	-
CIAV/IT/CK/1014-1/18	N	I	L	.	Q	Q	.	A	.	T	.	A	S
CIAV/IT/CK/1099/19	.	I	L	.	Q	Q	.	T	.	S	.	A	S
CIAV/IT/CK/1100/19	.	I	L	.	Q	Q	.	T	.	-	-	-	-
CIAV/IT/CK/1101/19	-	I	L	.	Q	Q	.	T	.	S	.	A	S
CIAV/IT/CK/1153-1/19	-	.	.	.	.	.	M	T	.	-	-	-	-
CIAV/IT/CK/1153-2/19	N	I	L	.	Q	Q	.	A	.	S	.	A	S
CIAV/IT/CK/1155/19	Q	.	.	.	Q	Q	.	.	.	R	.	.	S
CIAV/IT/CK/1157/19	N	I	L	.	Q	Q	.	A	.	T	.	A	S
CIAV/IT/CK/1179/19	-	I	L	.	Q	Q	.	T	.	-	-	-	-
CIAV/IT/CK/1180/19	Q	.	.	.	.	.	.	.	.	.	.	.	S
CIAV/IT/CK/1185/19	-	I	L	.	Q	Q	.	A	.	S	.	A	S
CIAV/IT/CK/1186/19	.	I	L	.	Q	Q	.	A	.	T	.	A	S
CIAV/IT/CK/1187/19	-	I	L	.	Q	Q	.	T	.	S	.	A	S
CIAV/IT/CK/1188/19	N	I	L	.	Q	Q	.	A	.	S	.	A	S
CIAV/IT/CK/1189/19	-	I	L	.	Q	Q	.	A	.	-	-	-	-
CIAV/IT/CK/1191/19	-	I	L	.	Q	Q	.	T	P	S	.	A	S
CIAV/IT/CK/1192/19	-	I	L	.	Q	Q	.	A	.	-	-	-	-
CIAV/IT/CK/1195/19	-	.	L	.	Q	Q	.	.	.	-	-	-	-
CIAV/IT/CK/1196/19	.	I	L	.	Q	Q	.	A	.	T	.	A	S
CIAV/IT/CK/1201/19	-	I	L	.	Q	Q	.	A	.	-	-	-	-

(.) = same amino acid of reference strain Del-Ros; (-) = sequence not available.

## Data Availability

The sequences generated in this study are available in GenBank under Accession numbers MT813068–MT813092.

## Supplementary Materials

The following are available online at https://www.mdpi.com/article/10.3390/ani11040944/s1, Table S1: reference strains retrieved from the GenBank database included in the recombination analysis.
