# Supplementary material for: Genetic Heterogeneity among Chicken Infectious Anemia Viruses Detected in Italian Fowl
Source: Animals (Basel). 2021 Mar 27;11(4):944. doi: 10.3390/ani11040944 (PMC8067058; doi:10.3390/ani11040944)
Supplement: Supplementary file 1 [file animals-11-00944-s001.pdf]

Table S1 *Reference strains retrieved from the GenBank databse included in the recombination analysis.*

CIAV strain	Country	GenBank Accession N°
Del-Ros	USA	AF313470
26P4	USA	D10068
Cux-1	Germany	M55918
Cuxhaven 1	Germany	M81223
CAU269-7	Australia	AF227982
3711	Australia	EF683159
BD-3	Bangladesh	AF395114
98D02152	USA	AF311892
Isolate 4	Taiwan	KJ728816
Isolate 6	Taiwan	KJ728817
Isolate 8	Taiwan	KJ728819
Isolate 7	Taiwan	KJ728818
Isolate 9	Taiwan	KJ728820
Isolate 18	Taiwan	KJ728827
Isolate 20	Taiwan	KJ728829
CAV-EG-2	Egypt	MH001553
CAV-EG-4	Egypt	MH001554
CAV-EG-6	Egypt	MH001555
CAV-EG-7	Egypt	MH001556
CAV-EG-10	Egypt	MH001557
CAV-EG-11	Egypt	MH001559
CAV-EG-13	Egypt	MH001560
CAV-EG-14	Egypt	MH001565
CAV-EG-15	Egypt	MH001568
CAV-EG-21	Egypt	MH001567
CAV-EG-23	Egypt	MH001562
CAV-EG-25	Egypt	MH001569
CAV-EG-26	Egypt	MH001564
CAV-EG-28	Egypt	MH001570
TR20	Japan	AB027470
N5	China	MK887167
G17.33.3	Vietnam	MH536104
HB1517	China	KU645516
AH6	China	DQ124935
HLJ15108	China	KY486137
LF4	China	AY839944
SD1514	China	KU645521
704	Australia	U65414
98D06073	USA	AF311900
1102PT01	Taiwan	KY888892
1103TN02	Taiwan	KY888894
SD22	China	DQ141673
SD24	China	AY999018
01-4201	USA	DQ991394
17JL0310	China	MK089241
3-1P60	Malaysia	AY040632
1504TW	Taiwan	MK358456
1535TW	Taiwan	MN299315

N.A. Not available

1637TW	Taiwan	MN299310
1852TW	Taiwan	MN299316
A2	Japan	AB031296
AH4	China	DQ124936
C14	China	EF176599
CAV/NAM/TANUVAS/09	India	KY053900
CAV-10	Argentina	KJ872513
CAV-18	Argentina	KJ872514
CAV-CA1-2015	Egypt	MG827098
CAV-SK4-2017	Egypt	MG827100
XH16	China	MK770259
N.A.	China	AF475908
CIAV89-69	South Korea	JF507715
CIAV-Mouse	China	KU645525
G17.33.3	Vietnam	MH536104
G6	Japan	AB119448
GD-102	China	KU050677
GD-K-12	China	KF224935
GX1904A	China	MN103402
GX1804	China	MK484615
HB1517	China	KU645516
HLJ14101	China	KY486136
HN1504	China	KU645512
JL14028	China	KY486148
LN1402	China	KU645511
LY-1	China	KX447636
SD1403	China	KU221054
SD1505	China	KU645523
SD1511	China	KU641015
SD1514	China	KU645521
SD1518	China	KU645522
SH11	China	DQ141670
SH16	China	DQ141671
HN9	China	DQ141672
TJBD33	China	AY843527
SDLY08	China	FJ172347
17SY0902	China	MK089243
SMSC-1	Malaysia	AF285882
SMSC-1P9WT	Malaysia	DQ217400
SMSC-1P123WT	Malaysia	DQ217401
